# Autophagy Deficiency by Atg4B Loss Leads to Metabolomic Alterations in Mice

**DOI:** 10.3390/metabo11080481

**Published:** 2021-07-27

**Authors:** Gemma G. Martínez-García, Raúl F. Pérez, Álvaro F. Fernández, Sylvere Durand, Guido Kroemer, Guillermo Mariño

**Affiliations:** 1 Departamento de Biología Funcional, Facultad de Medicina, Universidad de Oviedo, 33006 Oviedo, Spain; gema79gmg@gmail.com; 2 Instituto Universitario de Oncología (IUOPA), 33006 Oviedo, Spain; rauldiul@gmail.com; 3 Instituto de Investigación Sanitaria del Principado de Asturias (ISPA), 33011 Oviedo, Spain; ffernandezalvaro@gmail.com; 4 Cancer Epigenetics and Nanomedicine Laboratory, Nanomaterials and Nanotechnology Research Center (CINN-CSIC), 33940 El Entrego, Spain; 5 Departamento de Biología de Organismos y Sistemas (BOS), Facultad de Biología, Universidad de Oviedo, 33006 Oviedo, Spain; 6 Rare Diseases CIBER (CIBERER) of the Carlos III Health Institute (ISCIII), 28029 Madrid, Spain; 7 Departamento de Bioquímica y Biología Molecular, Facultad de Medicina, Universidad de Oviedo, 33006 Oviedo, Spain; 8 Centre de Recherche des Cordeliers, INSERM, U1138, F-75006 Paris, France; SYLVERE.DURAND@gustaveroussy.fr (S.D.); kroemer@orange.fr (G.K.); 9 Faculté de Médecine, Université Paris Descartes, Sorbonne Paris Cité, F-75006 Paris, France; 10 Pôle de Biologie, Hôpital Européen Georges Pompidou, AP-HP, F-75006 Paris, France; 11 Metabolomics and Cell Biology Platforms, Gustave Roussy Comprehensive Cancer Institute, F-94805 Villejuif, France

**Keywords:** autophagy, metabolome, ATG4, aging, autophagosome, ATG8, LC3, GABARAP, autophagin

## Abstract

Autophagy is an essential protective mechanism that allows mammalian cells to cope with a variety of stressors and contributes to maintaining cellular and tissue homeostasis. Due to these crucial roles and also to the fact that autophagy malfunction has been described in a wide range of pathologies, an increasing number of in vivo studies involving animal models targeting autophagy genes have been developed. In mammals, total autophagy inactivation is lethal, and constitutive knockout models lacking effectors of this route are not viable, which has hindered so far the analysis of the consequences of a systemic autophagy decline. Here, we take advantage of *atg4b^−/−^* mice, an autophagy-deficient model with only partial disruption of the process, to assess the effects of systemic reduction of autophagy on the metabolome. We describe for the first time the metabolic footprint of systemic autophagy decline, showing that impaired autophagy results in highly tissue-dependent alterations that are more accentuated in the skeletal muscle and plasma. These changes, which include changes in the levels of amino-acids, lipids, or nucleosides, sometimes resemble those that are frequently described in conditions like aging, obesity, or cardiac damage. We also discuss different hypotheses on how impaired autophagy may affect the metabolism of several tissues in mammals.

## 1. Introduction

Autophagy (from the Greek, “auto”: oneself and “phagy”: to eat) refers to any cellular degradative pathway that involves the delivery of cytoplasmic cargo to the lysosome [1,2]. The autophagic degradation routes can be classified into at least three different pathways: chaperone-mediated autophagy, microautophagy, and macroautophagy, which is the major lysosomal pathway for the turnover of cytoplasmic components and will be referred to simply as ‘autophagy’ hereafter. This process begins with the engulfment of cytoplasmic constituents by a membrane sac, called the isolation membrane or phagophore. Then, this structure forms a double-membrane vesicle, termed the autophagosome, which may contain either bulk or selected portions of cytoplasm [3]. Once their formation is complete, autophagosomes eventually fuse with lysosomes and acquire hydrolytic activity. Finally, the autophagic cargo is degraded, and the resulting essential biomolecules are recycled back to the cytoplasm to satisfy the anabolic and energetic requirements of the cell. Autophagic activity can be enhanced in response to a wide variety of intracellular and extracellular stimuli and represents an essential mechanism by which organisms can adapt to acute stress conditions [4].

In lower eukaryotes such as yeast, autophagy is mainly involved in adaptation to starvation, constituting a resistance pathway aimed at reallocating resources from growth towards somatic preservation when nutrients are scarce. In multicellular organisms, autophagy has evolved as a multifunctional pathway involved in a variety of additional processes. In mammals, autophagy constitutes a major protective mechanism that allows cells to cope with different types of stressors and that helps defend the organism against degenerative, inflammatory, infectious, and neoplastic diseases [1]. Disabled autophagy is directly involved in the pathogenesis of multiple diseases including cancer, viral infection, chronic inflammatory pathologies, and a variety of neurodegenerative disorders. Conversely, an increase in autophagic activity can enhance the resistance of cells to multiple distinct kinds of stress, even at the whole-body level [4].

In mice, the knockout of several mammalian autophagy genes, such as *Atg3*, *Atg5*, *Atg7*, *Ambra1*, *FIP200,* and *Becn1*, among others, causes a complete (and lethal) defect in autophagy. This is because these genes operate in a non-redundant way in central hubs of the autophagic pathway [5]. This characteristic has largely precluded the study of the effects of a reduction in systemic autophagy. In contrast to the former cases, mammals possess several orthologs of yeast Atg4 protease that together form the ATG4 protein family, composed of ATG4A, ATG4B, ATG4C, and ATG4D [6]. Among them, ATG4B has been shown to be the most important ATG4 protease in mammalian cells and tissues. However, because there is a certain degree of functional redundancy in the ATG4 protein family, ATG4B loss in mice is compatible with a close-to-normal development before and beyond birth [7]. Despite being viable, *atg4b^−/−^* mice exhibit a significant reduction of their autophagic potential in multiple distinct organs. Thus, autophagic flux, as well as the depletion of the autophagic substrate SQSTM1/p62, is diminished, but not totally impaired, in both fed and starved *atg4b^−/−^* mice compared with wild-type (*WT*) littermates [7]. Due to these specific characteristics, *atg4b^−/−^* mice have been successfully used in a wide variety of studies to address the role of autophagy in multiple pathological and physiological processes [7,8,9,10,11,12,13,14].

Here, we describe for the first time the metabolic footprint of systemic reduced autophagy in laboratory mice. Through mass spectrometry-based metabolite profiling of skeletal muscle, heart, and liver tissues and also plasma from age-matched WT and *atg4b^−/−^* mice, we describe and discuss the alterations found in their metabolomic profiles. We also provide hypotheses on how reduced autophagy might impact the activity of different metabolic pathways in different tissues in mammals.

## 2. Results and Discussion

### 2.1. Metabolomic Profiling of Autophagy-Deficient Mouse Tissues

**Figure 1:**
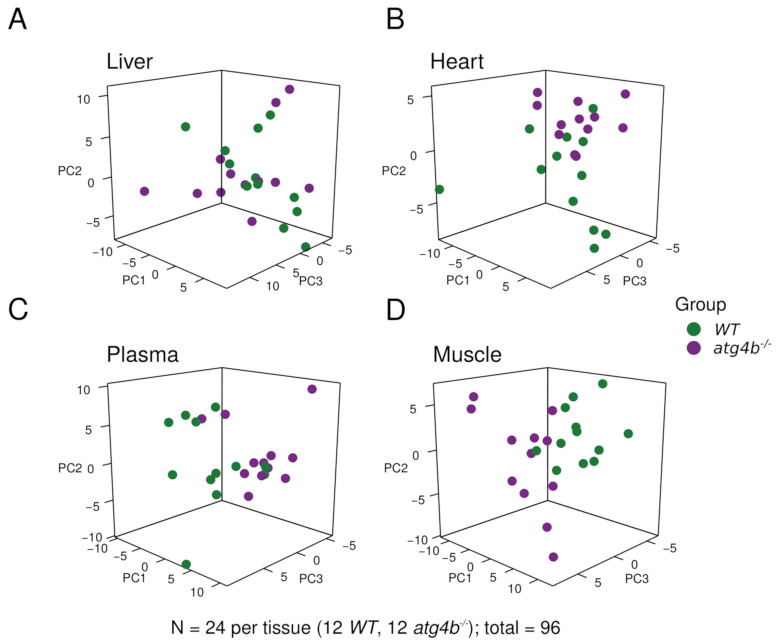
Metabolomic profiling of autophagy-deficient mouse tissues. Principal component analysis plots showing the distribution of WT and *atg4b*^−/−^ samples in liver (**A**), heart (**B**), plasma (**C**), and skeletal muscle tissue (**D**) across the first three principal components.

In order to avoid fluctuations in tissue metabolite concentration due to differential feeding habits among individual mice, feeding was synchronized. Thus, a starvation/feeding regimen was conducted in all mice of the analyzed experimental groups, as previously described [15]. First, mice were fasted overnight and then fed a pelleted laboratory diet for 3 h. Subsequently, food was removed to avoid re-feeding, and mice were euthanatized 6 h later in order to extract the plasma, liver, heart, and quadriceps (as a representative skeletal muscle) tissue samples. We performed our analyses in these tissues as heart, liver, and skeletal muscle require autophagic degradation to efficiently perform their functions [16], which are essential for mammalian biology. Samples were flash-frozen and processed according to standard procedures for metabolite extraction and subjected to chromatography and mass spectrometry, in both open profiling and targeted modes [17]. This approach was chosen to enable the monitoring of well-known metabolites as well as others not so frequently assessed in biological studies, which could be important for our experimental setting. By using this approach, we were able to detect 143, 130, 164, and 121 bona fide, precisely identified metabolites in skeletal muscle, heart, liver, and plasma, respectively (Figure 1 and Supplementary Table S1). After data pre-processing, principal component analyses (PCAs) of the metabolomic profiles revealed that the degree of autophagy deficiency caused by ATG4B loss impacted global abundances of metabolites to a variable degree, depending on the analyzed tissue. As shown in Figure 1, although autophagy deficiency affected liver and heart metabolomes, we could also observe substantial inter-individual variability, despite our efforts regarding feeding synchronization. In contrast, inter-individual variability was substantially reduced for plasma and skeletal muscle samples, in which the autophagy defect of *atg4b*^−/−^ mice caused a significant alteration in the metabolomic profiles (Figure 1).

### 2.2. Autophagy Deficiency upon ATG4B Loss Leads to Alterations in Amino Acid and Fatty Acid Metabolism of the Skeletal Muscle

**Figure 2:**
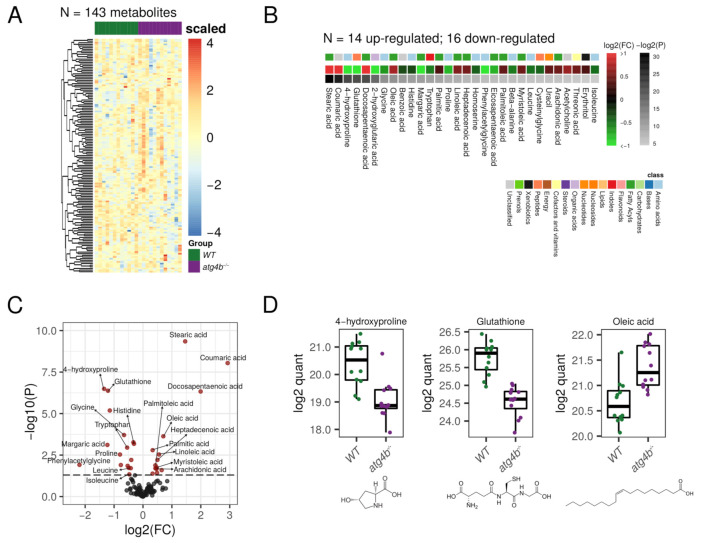
Autophagy deficiency upon ATG4B loss in skeletal muscle. (**A**) Heatmap showing the row-scaled log2 quantification levels of all the profiled metabolites in skeletal muscle tissue samples. (**B**) Heatmap describing the change (log2 of fold change) of the metabolites with significantly altered levels between WT and *atg4b*^−/−^ samples. (**C**) Volcano plot comparing the effect size (log2 of fold change) to the significance (−log10 of *p*-value) of the metabolites altered between WT and *atg4b^−/−^* samples. Significant metabolites are colored in red. Representative metabolites are labeled within the plot. (**D**) Boxplots showing the log2 quantification levels of three representative metabolites, which are significantly altered between WT and *atg4b^−/−^* samples. Significance is defined as an unadjusted *p*-value < 0.05.

In our metabolomic profiling of WT and *atg4b*^−/−^ mice skeletal muscle extracts (quadriceps), we were able to detect 143 bona fide non-redundant metabolites, of which 30 were differential metabolites (DMs; see Supplementary Table S1). Five of these DMs corresponded to amino acids and other three to amino acid derivates or amino acid synthesis intermediates. As shown in Figure 2, *atg4b*^−/−^ mice muscle extracts had reduced levels of the essential amino acids leucine, histidine, and tryptophan, as well as the semi-essential amino acids glycine and proline, as compared to their age-matched WT controls. This is consistent with the essential role of the autophagy pathway as a one of the main cellular suppliers of amino acids [15,18]. The reduced levels of these amino acids might impact muscle cells’ metabolic circuitry, due to the importance of some of these amino acids for the main metabolic cellular pathways.

Among the essential amino acids, leucine, a branched-chain amino acid (BCAA), is probably the most biologically active and is involved in the regulation of a variety of cellular processes. For example, leucine positively regulates the activity of mammalian target of rapamycin (mTOR), key regulator of protein synthesis, among other processes [19,20]. Leucine also upregulates a variety of other important processes for cellular metabolism, such as glucose uptake, mitochondrial biogenesis, or fatty acid oxidation [21,22]. This amino-acid can be used to obtain Acetyl-CoA, which could be incorporated into the tricarboxylic acid (TCA) cycle and is also involved in autophagy regulation [23]. Histidine is a semi-essential proteinogenic amino acid, which can also be used to synthesize other amino acids, such as glutamic and aspartic acids or important biomolecules as histamine, with high histidine levels being associated with growth and tissue repairing [24]. Tryptophan has been recently described to regulate skeletal muscle mass, and low serum levels of this amino acid have been associated with skeletal muscle atrophy [25]. *atg4b^−/−^* mice also showed reduced levels of glycine, another semi-essential amino acid that has profound inhibitory effects on inflammatory cell activation and inflammatory processes [26]. Additionally, the levels of homoserine, an intermediate amino acid in the synthesis of methionine, threonine, and isoleucine, were also significantly lower in autophagy-deficient mice. Similarly, proline and its hydroxylation product 4-hydroxyproline were also reduced in skeletal muscle extracts from *atg4b^−/−^* mice. Hydroxyprolines are one of the main components of collagen fibers and constitute the main traditional markers of bone resorption [27]. The observed decrease in hydroxyprolines levels may indicate the breakdown of muscle fibers in conditions of autophagy deficiency, such as that of *atg4b^−/−^* mice.

Interestingly, autophagy deficiency strongly modified the muscle lipidic profile in mice. In fact, out of the 30 DMs found in this tissue, 11 corresponded to fatty acids and 2 to fatty acid-related metabolites. In *atg4b^−/−^* mice muscle extracts, there was a significant increase in the concentration of several saturated (such as stearic or palmitic acids), monounsaturated (such as oleic, palmitoleic, myristoliecic, or heptadecenoic acids), and polyunsaturated (such as arachidonic, linoleic, docosapentaenoic, or eicosapentaenoic) fatty acids (Figure 2). Intriguingly, an increase in fatty acid content in skeletal muscle is associated with aging, with many of the fatty acids that were increased in the skeletal muscle of *atg4b^−/−^* (such as palmitic, oleic, heptadecenoic, linoleic, or docosapentaenoic) also being increased in muscles from aged WT mice [28]. Additionally, phenylacetylglycine, a metabolite involved in fatty acids synthesis, was reduced in *atg4b^−/−^* mice muscle extracts.

Among several others, another important metabolite like glutathione (GSH) was also significantly reduced in *atg4b^−/−^* mice muscles (Supplementary Table S1). A reduction in this antioxidant, which is involved in the protection against oxidative stress and in the regulation of protein thiolation, has been documented in skeletal muscles from aged laboratory mice [28]. Interestingly, calorie restriction (CR), the most successful strategy employed to delay aging in all species tested so far, from lower eukaryotes (yeast) to rodents (mice) or even primates (Rhesus monkeys) [29], has been shown to reverse this effect [30].

### 2.3. Autophagy Deficiency upon ATG4B Loss Leads to Alterations in Nitrogenous Bases and Nucleotides Metabolism of the Heart

**Figure 3:**
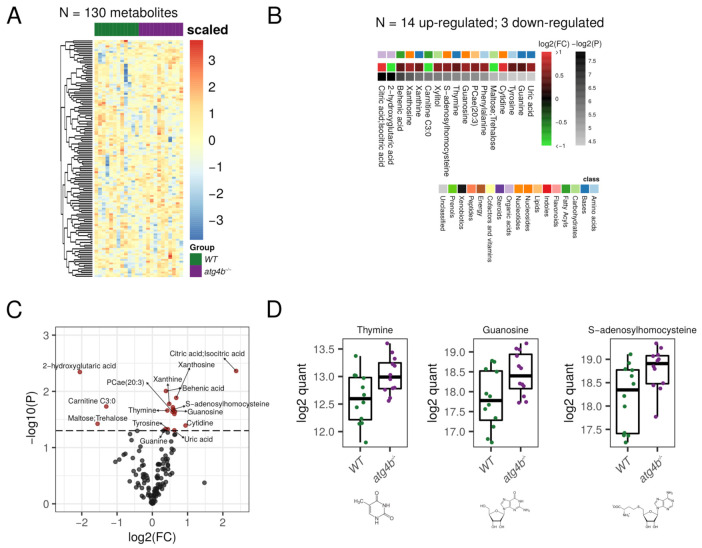
Autophagy deficiency upon ATG4B loss in heart. (**A**) Heatmap showing the row-scaled log2 quantification levels of all the profiled metabolites in heart tissue samples. (**B**) Heatmap describing the change (log2 of fold change) of the metabolites with significantly altered levels between WT and *atg4b^−/−^* samples. (**C**) Volcano plot comparing the effect size (log2 of fold change) to the significance (−log10 of *p*-value) of the metabolites altered between WT and *atg4b^−/−^* samples. Significant metabolites are colored in red. Representative metabolites are labeled within the plot. (**D**) Boxplots showing the log2 quantification levels of three representative metabolites, which are significantly altered between WT and *atg4b^−/−^* samples. Significance is defined as an unadjusted *p*-value < 0.05.

In heart extracts, we were able to detect 130 bona fide non-redundant metabolites in our profiling. Among them, 17 were differential metabolites (DMs) for *atg4b^−/−^* mice (Supplementary Table S1). Four of these DMs, which were increased in the heart metabolome of autophagy-deficient mice, corresponded to purine/pyrimidine bases (thymine, guanine, and xantine) and three to nucleosides (guanosine, cytidine, and xanthosine). The transcriptional shift of genes involved in purine biosynthesis pathways and in nucleotide catabolism has been documented during pathological cardiac remodeling in the context of Huntington’s-disease-related cardiomyopathy [31]. The fact that Huntington’s disease is characterized by autophagy dysfunction [32] and that inhibition of autophagy in the heart induces age-related cardiomyopathy [33] suggests that the observed shift in nucleotide metabolism in *atg4b^−/−^* mice could be a sign of ongoing cardiac remodeling towards hypertrophy. In line with these results, we detected an increase in the concentration of uric acid, the major catabolite of cardiac adenine nucleotides and adenosine [34] in *atg4b^−/−^* hearts. Interestingly, increased levels of uric acid have been found in the heart after cardiac damage elicited by a variety of experimental approaches, including acute myocardial ischemia in laboratory rats [35] or doxorubicin-induced cardiotoxicity in mice [36]. Conversely, the levels of ribose-5-phosphate, the main precursor in nucleotide biosynthetic pathways, were decreased in samples from *atg4b^−/−^* mice (Figure 3). This ribose-5-phosphate depletion could be a consequence of an upregulation of de novo synthesis of nucleosides, which could account for their observed increase in heart extracts from *atg4b^−/−^* mice. Altogether, these results show that cardiac autophagy deficiency in laboratory mice leads to alterations in nitrogen bases and nucleoside metabolism.

In addition to these alterations, autophagy-deficient mice also showed an increase in cardiac levels of the metabolite S-adenosylhomocysteine (SAH). This metabolite is, simultaneously, a by-product and a potent inhibitor of the activity of SAM-dependent methyltransferases [37], with high SAH levels being associated with DNA hypomethylation [38]. Interestingly, a rise in SAH levels has been widely associated with an increased risk of cardiovascular diseases [39]. In fruit flies, tissue content of SAH increases with aging, with lower levels of this metabolite being found in long-lived strains of fruit flies. Interestingly, down-regulation of S-adenosyl-homocysteine in genetically engineered flies extends health-span and life-span in this model organism [40].

Moreover, *atg4b^−/−^* hearts also showed an increase in the concentration of several fatty acids such as the saturated behenic acid, with similar trends being observed for the polyunsaturated docosapentaenoic and arachidonic acids, which is consistent with the observed increase in fatty acids in muscle extracts from autophagy-deficient mice. Other significantly altered metabolites in *atg4b^−/−^* heart extracts were the phospholipid phosphatidylcholine 20:3, the amino acids phenylalanine and tyrosine, the TCA intermediate citric acid, and the sugar xylitol, which is an endogenous metabolite of the uronic acid cycle.

### 2.4. Autophagy Deficiency upon ATG4B Loss Has a Minor Impact in Liver Tissue Metabolome

**Figure 4:**
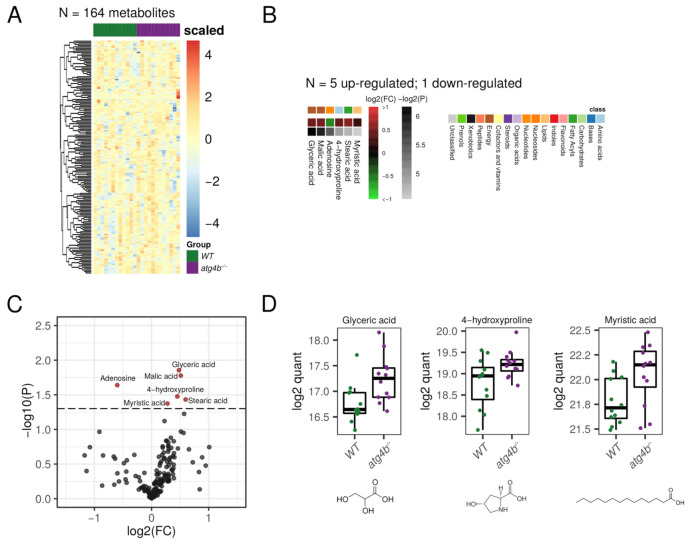
Autophagy deficiency upon ATG4B loss in liver. (**A**) Heatmap showing the row-scaled log2 quantification levels of all the profiled metabolites in liver tissue samples. (**B**) Heatmap describing the change (log2 of fold change) of the metabolites with significantly altered levels between WT and *atg4b^−/−^* samples. (**C**) Volcano plot comparing the effect size (log2 of fold change) to the significance (−log10 of *p*-value) of the metabolites altered between WT and *atg4b^−/−^* samples. Significant metabolites are colored in red. Representative metabolites are labeled within the plot. (**D**) Boxplots showing the log2 quantification levels of three representative metabolites, which are significantly altered between WT and *atg4b^−/−^* samples. Significance is defined as an unadjusted *p*-value < 0.05.

In our metabolomics profiling of liver extracts, we were able to detect 164 bona fide non-redundant metabolites. Surprisingly, among all of them, only six metabolites were significantly altered in *atg4b^−/−^* mice (Figure 4, Supplementary Table S1). Specifically, the saturated fatty acid myristic acid, the Krebs cycle intermediate malic acid, the proline hydroxylation product 4-hydroxyproline, and glyceric acid were up-regulated in *atg4b^−/−^* mice. Conversely, only adenosine levels were found to be significantly reduced in these animals.

### 2.5. Metabolomic Alterations Caused by Autophagy Deficiency in Plasma

**Figure 5:**
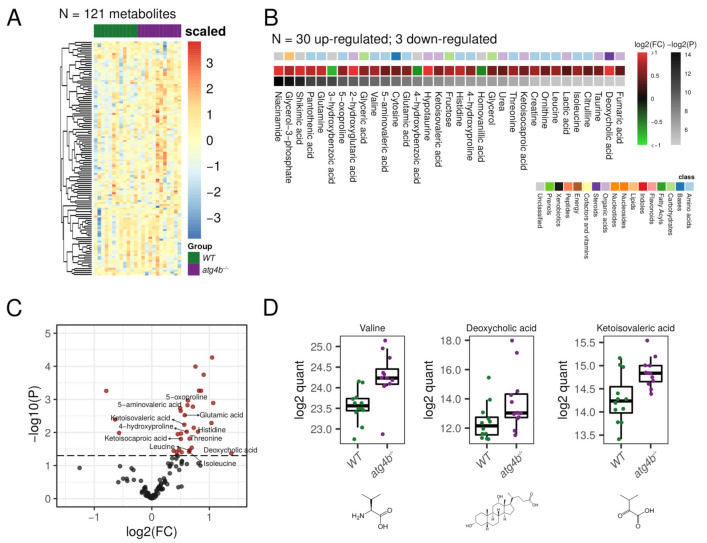
Autophagy deficiency upon ATG4B loss in plasma. (**A**) Heatmap showing the row-scaled log2 quantification levels of all the profiled metabolites in plasma samples. (**B**) Heatmap describing the change (log2 of fold change) of the metabolites with significantly altered levels between WT and *atg4b^−/−^* samples. (**C**) Volcano plot comparing the effect size (log2 of fold change) to the significance (−log10 of *p*-value) of the metabolites altered between WT and *atg4b^−/−^* samples. Significant metabolites are colored in red. Representative metabolites are labeled within the plot. (**D**) Boxplots showing the log2 quantification levels of three representative metabolites, which are significantly altered between WT and *atg4b^−/−^* samples. Significance is defined as an unadjusted *p*-value < 0.05.

In our metabolomic profiling of plasma, we were able to detect 121 bona fide non-redundant metabolites, 33 of them being differential metabolites (DMs) between WT and *atg4b^−/−^* mice (Figure 5, Supplementary Table S1). Among DMs that were increased in the plasma metabolome of autophagy-deficient mice, nine matched to amino acids (leucine, isoleucine valine, glutamic acid, glutamine, histidine, threonine, ornithine, and citrulline) and five to amino acid derivates (ketoisocaproic acid, ketoisovaleric acid, 5-oxoproline, 4-hydroxyproline, and 5-aminovaleric acid). Interestingly, high circulating levels of valine, leucine, and isoleucine have been reported to increase the risk of developing diabetes mellitus [41,42]. Moreover, high circulating citrulline levels in mice are proposed to predict the development of the metabolic syndrome [43]. Consistently, increased plasma BCAAs, citrulline, and ornithine levels are found in mice subjected to streptozotocin administration (STZ), which leads to the development of type I diabetes by loss of beta-cell pancreatic islets [43]. These alterations could contribute to explain the increased susceptibility of *atg4b^−/−^* mice to developing both type I and type II diabetes [44]. In addition to all three branched-chain amino acids (BCAAs), the keto acids keto-isocaproic and keto-isovaleric acid were also increased in plasma from *atg4b^−/−^* mice. Interestingly, high BCAAs and their keto acids, BCKAs, are typical of maple syrup disease and can also be found in the murine model of the disease, Ppm1k-deficient mice [45]. Although the increases in circulating levels of BCAAs and BCKAs in *atg4b^−/−^* mice are not as high as those found in individuals severely affected by maple syrup disease, raw values of leucine and valine average levels in *atg4b^−/−^* mice plasma were increased by 1.67 and 1.69, respectively, similar to levels found in patients suffering from mild maple syrup urine disease (MSUD) [46].

MSUD is caused by a deficiency of the branched-chain alpha-keto acid dehydrogenase complex (BCKDC), which is essential for branched-chain amino acids catabolism. Although a direct connection between autophagy and BCKDC function has not been established, keto-isocaproic acid has been shown to inhibit autophagic flux in vivo in a BCKDC-dependent fashion [47]. As BCAAs and BCKAs accumulation is one of the main effectors of MSUD-associated pathologies, *atg4b^−/−^* mice might be predisposed to the development of pathologies typically found in patients suffering mild forms of MSUD. In this sense, one of the main metabolic disturbances in MSUD patients is metabolic acidosis [48]. Interestingly, *atg4b^−/−^* mice show elevated circulating levels of the glutamic acid derivate 5-oxoproline, whose increase is associated with metabolic acidosis upon chronic acetaminophen use [49]. Another amino acid derivate that was found to be increased in *atg4b^−/−^* mice plasma is 4-hydroxyproline. A rise in the levels of circulating hydroxyprolines is a marker of bone-associated pathologies like Paget disease [50] and is also associated with a higher incidence of connective tissue injuries [51], as well as liver damage and fibrosis [52]. Interestingly, this metabolite is also increased in liver and decreased in skeletal muscle extracts from autophagy-deficient mice, as mentioned above.

Among the metabolites whose levels were most dramatically increased in autophagy-deficient mice was unconjugated deoxycholic acid, one of the most biologically active bile acids. Moreover, ursodeoxycholic acid, another important bile acid, also tended to be increased in mutant mice plasma. Apart from their functions in lipid digestion, bile acids also act as hormones involved in the regulation of various metabolic processes (including triglyceride, cholesterol, glucose, and energy homeostasis) through the activation of the farnesoid X receptor (FXR) and Takeda G-Protein-Coupled Receptor 5 (TGR5), among others [53]. Although some studies suggest that bile acids may protect from diet-induced obesity by upregulating brown fat thermogenesis [54,55], increased circulating levels of unconjugated bile acids, including deoxycholic acid, have been shown to induce skeletal muscle atrophy in mice [56]. Moreover, patients suffering from non-alcoholic fatty liver disease (NAFLD) show increased levels of circulating unconjugated bile acids [57], which is consistent with the increased propensity to develop diet- and STZ-hepatic steatosis of *atg4b^−/−^* mice [44].

### 2.6. Metabolic Pathways Affected by Authophagy Deficiency Are Tissue-Dependent

**Figure 6:**
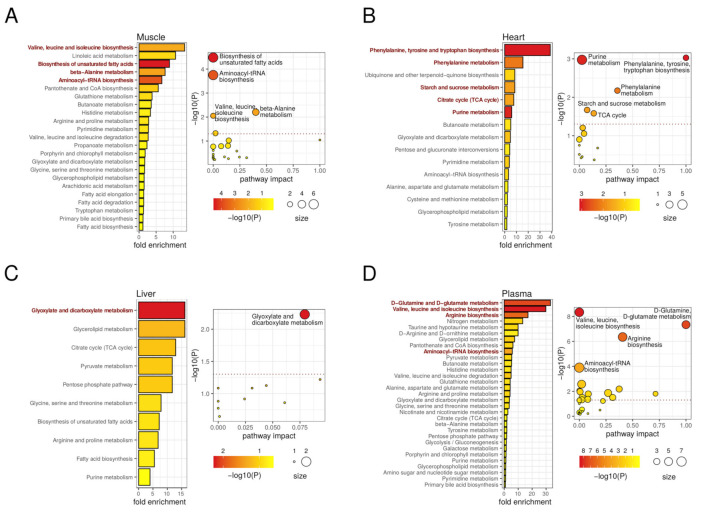
Metabolic pathways involved in autophagy deficiency upon ATG4B loss in mouse tissues. On the left, the bar plots describe altered metabolic pathways with detected fold-enrichment, colored by significance (−log10 of *p*-value), across differential metabolites for the muscle (**A**), heart (**B**), liver (**C**), and plasma (**D**) comparisons. On the right, the scatterplots describe the pathway impact, size, and significance of the detected metabolic pathways for muscle (**A**), heart (**B**), liver (**C**), and plasma (**D**). The horizontal dotted line in the scatterplots marks the 0.05 *p*-value threshold (unadjusted). Representative significant (unadjusted *p*-value < 0.05) pathways are highlighted in the bar plots and labeled in the scatterplots.

Our metabolomics analyses in autophagy-deficient mice revealed a variety of DMs, which were normally tissue-specific. With all these data, we performed pathway enrichment analyses using MetaboAnalyst [58]. These analyses led to the identification of different metabolic pathways that were significantly affected by the autophagy defect of *atg4b^−/−^* mice in plasma and in the different tissues of our analyses (Figure 6).

In skeletal muscle, we observed an enrichment of metabolic pathways involving different amino acids, such as valine, leucine, and isoleucine biosynthesis, as well as beta-alanine metabolism and aminoacyl-tRNA biosynthesis, in muscles from *atg4b^−/−^* mice (Figure 6A). In addition, some metabolic pathways involved in fatty-acid metabolism, such as biosynthesis of unsaturated fatty acids, were also enriched in mutant mice. In heart tissue, the analysis revealed an enrichment in pathways that are associated with upregulated metabolites, such as phenylalanine, tyrosine, and tryptophan biosynthesis, phenylalanine metabolism, purine metabolism, and TCA cycle, whereas a downregulation of starch and sucrose metabolism was observed in *atg4b^−/−^* mice (Figure 6B). The minor impact of ATG4B deficiency in liver tissue transduced in the detection of fewer metabolic pathways, mainly the glyoxylate and dicarboxylate metabolism pathway (Figure 6C). By contrast, a variety of metabolic pathways were altered in *atg4b^−/−^* mice plasma. Among them, those associated with upregulated amino acids such as upregulation of valine, leucine, and isoleucine biosynthesis; D-glutamine and D-glutamate metabolism; arginine biosynthesis or aminoacyl-tRNA biosynthesis showed high statistical significance (Figure 6D). It is remarkable that many of these metabolites were significantly downregulated in skeletal muscle of autophagy-deficient mice. Moreover, the increased presence of BCAAs in *atg4b^−/−^* mice plasma contrasts with some previous reports showing a reduction in circulating BCAAs levels in animal models of total systemic autophagy impairment [18]. The fact that *atg4b^−/−^* mice show a partial autophagy deficiency could explain this apparently contradictory result. In this sense, it is likely that a partial autophagy decrease (but not its total impairment) is able to maintain appropriate circulating BCAA levels, which would increase up to the values observed in *atg4b^−/−^* mice as a consequence of additional alterations due to ATG4B loss that might be at odds with those found upon total loss of autophagic degradative potential.

## 3. Materials and Methods

### 3.1. Tissue Samples Preparation for Metabolomics Analyses

Tissues were extracted from mice immediately after sacrifice and frozen in liquid nitrogen. Tissue samples were then kept at −80 °C until tissue homogenization. For metabolites extraction, tissue fragments of 30 mg were put in 300 µL cold lysis buffer (methanol:water:chloroform, 9:1:1, −20 °C) and homogenized with a Precellys 24 Homogenizer (Bertin Technologies, Montigny-le-Bretonneux, France), according to standard protocols. Tissue extracts were centrifuged for 10 min (15,000× *g*, 4 °C), and supernatants (150 µL) were collected in microcentrifuge tubes. Serum aliquots of 100 µL were mixed with 500 µL cold solvent (methanol:water:chloroform, 9:1:1, −20 °C) in microcentrifuge tubes, vortexed, and centrifuged for 10 min (15,000× *g*, 4 °C). Both tissue and serum samples were then dried at 40 °C in Techne DB3 Dri-Block-heater (Bibby Scientific Ltd., Stone, UK). On the day of analysis, dried extracts were resuspended in 300 µL of methanol and split in equal parts for liquid chromatography–mass spectrometry (LC-MS) and gas chromatography–mass spectrometry (GC-MS) analyses. GC-MS samples were transferred into glass tubes, and 10 µL aliquots were destined to quality control (QC) before evaporation and chemical derivatization. LC-MS samples were dried again, resuspended in 300 µL water, and split as follows: 2 × 50 µL aliquots were transferred to HPLC vials for targeted (LC-QQQ) and profiling (LC-QTOF) analysis, 1 × 10 µL aliquot was destined to QC, and the remaining material was kept as back-up. For all analyses, acquisition was performed randomly alongside five QC samples injected at regular intervals. The instrument was calibrated daily with pre-validated standard solutions and the auto-tuning function.

### 3.2. Targeted Analysis by GC Coupled to Triple Quadrupole (QQQ) Mass Spectrometry

Dried extracts were dissolved in 50 µL of pyridine (Sigma-Aldrich, Saint Louis, MO, USA) supplemented with 20 mg/L methoxyamine hydrochloride (Sigma-Aldrich, Saint Louis, MO, USA) and left at room temperature in the dark. Sixteen hours later, 80 µL N-methyl-N-(trimethyl-silyl)trifluoroacetamide (MSTFA, Sigma-Aldrich, Sigma-Aldrich, Saint Louis, MO, USA) was added to the samples, and the latter were incubated for 30 min at 40 °C. GC studies were conducted as previously described [59]. GC-MS/MS acquisitions were performed on a 7890A gas chromatograph coupled to a triple quadrupole 7000 A detector (both from Agilent Technologies, Santa Clara, CA, USA), equipped with an electronic impact source (EIS) operating in positive mode and a 30 m × 0.25 mm I.D. × 0.25 mm film thickness HP5MS capillary column (Agilent Technologies, Santa Clara, CA, USA). Sample aliquots of 1 µL were injected into an inlet operating in splitless mode and set at 250 °C. Helium gas flow rate was set at 1 mL/min and the septum purge flow at 3 mL/min. The temperature was programmed as follows: 60 °C for 1 min, +10 °C/min up to 210 °C, hold for 3 min, +5 °C/min up to 325 °C, and hold for 5 min. The transfer line and ion-source temperatures were 250 and 230 °C, respectively. The duty cycle was 39 min. In all, 267 MRM transitions corresponding to 118 analytes were quantified with the MassHunter Quantitative Analysis software (Agilent Technologies, B.05.00), and results were exported to the R statistical environment for data reduction and statistical analyses.

### 3.3. Untargeted Analysis by UHPLC Coupled to Quadrupole-Time of Flight (QTOF) Mass Spectrometry

Profiling of tissue/serum metabolites was performed on a RRLC 1260 system (Agilent Technologies, Santa Clara, CA, USA) coupled to a QTOF 6520 detector (Agilent Technologies, Santa Clara, CA, USA), equipped with an electrospray source operating in full scan mode, from 50 to 1000 Da for both positive and negative ionization modes. The gas temperature was set at 350 °C, gas flow of 12 L/min, capillary voltage at 3.5 kV, and fragmentor voltage at 120 V. Two reference masses were used to maintain the mass accuracy during analysis: *m*/*z* 121.050873 and *m*/*z* 922.009798 in positive mode, and *m*/*z* 112.985587 and *m*/*z* 980.016375 in negative mode. Sample aliquots of 10 mL were injected on a Sb-Aq column (100 × 2.1 mm, particle size 1.8 mm, Agilent Technologies, Santa Clara, CA, USA), protected by a XDB-C18 guard column (5 × 2.1 mm, particle size 1.8 mm, Agilent Technologies) and heated at 40 °C. The gradient mobile phase consisted of 0.2% acetic acid (v:v in water) (A) and acetonitrile (B). The flow rate was set at 0.3 mL/min. The initial condition was set as 98% phase A and 2% phase B, and the gradient changes as follows: from 2% to 95% phase B in 7 min, 95% phase B for 3 min, and equilibration with 2% phase B for 3 min. The autosampler was kept at 4 °C. Profiling data were treated as described below.

### 3.4. Targeted Analysis by UHPLC Coupled to Triple Quadrupole (QQQ) Mass Spectrometry

Targeted analysis was performed on a RRLC 1260 system coupled to a Triple Quadrupole 6410 detector (Agilent Technologies, Santa Clara, CA, USA), equipped with an electrospray source operating in positive mode. Gas temperature was set at 350 °C, gas flow at 12 L/min, and capillary voltage at 3.5 kV. Sample aliquots of 10 mL were injected on a Zorbax Eclipse XDB-C18 column (100 × 2.1 mm, particle size 1.8 mm, Agilent Technologies, Santa Clara, CA, USA), protected by a XDB-C18 guard column (5 × 2.1 mm, particle size 1.8 mm) and heated at 40 °C. The gradient mobile phase consisted of 2 mM of dibutyl ammonium acetate (DBAA) in water (A) and acetonitrile (B). The flow rate was set at 0.2 mL/min, and the gradient changed as follows: initial condition (90% phase A and 10% phase B) was maintained for 4 min, from 10% to 95% phase B over 3 min. The column was washed using 95% mobile phase B for 3 min and equilibrated using 10% phase B for 3 min. The autosampler was kept at 4 °C. Target and qualified MRM transitions corresponding to 13 metabolites (adenosine, Adenosine monophosphate (AMP), Adenosine diphosphate (ADP), Adenosine triphosphate (ATP), Nicotinamide adenine dinucleotide (NAD), Nicotinamide adenine dinucleotide phosphate (NADP), nicotinamide adenine dinucleotide + hydrogen (NADH), Nicotinamide adenine dinucleotide phosphate + hydrogen (NADPH), flavin adenine dinucleotide (FAD), acetyl-CoA, malonyl-CoA, succinyl-CoA, and coenzyme A) were quantified with the MassHunter Quantitative Analysis software (B.04.00) and the results were exported to the R statistical environment for data reduction and statistical analyses.

### 3.5. Signal Processing for LC-QTOF Profiles

Profiles generated by LC-QTOF were processed using an in-house set of tools that convert raw MS data into a matrix compatible with statistical analysis. Raw data files were treated with the molecular feature extraction (MFE) algorithm of the MassHunter Quantitative Analysis software to identity predominant ions in form of triplets (mass to charge ratio (*m*/*z*); retention time (RT); intensity). Ions (1) that were flagged as isotopes by the MFE algorithm, (2) that had a mass defect between 0.75 and 0.95, (3) with a signal intensity below 3000, and (4) outside the 0.8–8 min RT range were discarded from downstream processing. To circumvent the elevated false-negative rate achieved by the vendor MFE algorithm, in-house scripts were used to extract, filter, align, and integrate the ion chromatograms from the MFE feature lists. In short: (1) feature lists were roughly grouped across all the samples; (2) for each cluster, extracted ion chromatograms (EICs) were generated with a tolerance of 16 ppm and 0.5 min; (3) EICs of poor quality (spikes and/or high-frequency noise) or detected in less than two samples were excluded; (4) high-quality EICs found in the QC samples were employed to perform time–domain alignment; and (5) peaks were identified and integrated across all samples. Peaks detected by LC-QTOF were classified into three annotation categories after interrogating our in-house annotation database MetIGR with each peak feature *m*/*z* and RT. “MetIGR” aggregates chemical entities taken from KEGG, HMDB, LipidMaps, ChEBI, and MetaCyc and implemented as previously described [60] and contains information related to biochemical pathways, drug modes of action, several ontologies, as well as the analytical characteristics of >200 standards of pure compounds measured in the exact conditions as the experimental samples were. So-called “annotated” peaks have their identity provisionally confirmed by matching both accurate mass and RT (within 17 ppm and 10 s windows) to a chemical standard. Please note that the RT for most acyl-carnitines and fatty acids were inferred from their respective number of carbons and double bonds. The accurate mass of so-called “putative” features matches (+/−17 ppm) either the deprotonated ([M-H]^−^) or the protonated ([M+H]^+^) ionization product of a MetIGR entry that is recorded as endogenous in HMDB, with a biological role in KEGG/BRITE or employed in RECON. Finally, the remaining signals found by the deconvolution algorithm were labelled as “profiling” features. The origin of these features is largely unknown. Based on accurate mass, some of the profiling features might (1) have matched an entry in our library of standards but failed the RT criterion, (2) have matched an entry in MetIGR that is not classified as a molecule of potential interest, or (3) be a less frequent ionization product of a MetIGR entry. LC-QTOF profile data are presented as follows: annotated compounds are denoted with their common name, putative features with the empirical formula of the metabolite in its neutral form, and profiling features with their accurate mass. For the three categories, names are followed by the retention time and measurement conditions (i.e., positive, *p*, or negative, n).

### 3.6. Metabolite Data Preprocessing and Statistical Analyses

Peaks extracted from LC-QTOF profiling or targeted studies were gathered and further reduced before data analysis and interpretation. Data were preprocessed and analyzed using R statistical software (v3.6.2). Normalized area intensity levels were log2-transformed prior to analyses. Missing data were imputed by half of the minimum value and metabolites with missing data in 25% or more samples were filtered out. A total of 143, 130, 164, and 121 metabolites were finally analyzed for skeletal muscle, heart, liver, and plasma samples, respectively, with a total of 223 unique metabolites being detected across all the tissues. Differential metabolites were called between *WT* and *atg4b^−/−^* subjects by using empirical Bayes moderated t-tests in a linear model framework (*limma* v3.42.2) including sex as a covariate [61]. Due to the high variability encountered, metabolites with unadjusted *p*-values < 0.05 were considered statistically significant to allow a more complete exploration of the results (see Supplementary Table S1 for the full results of the differential analyses). Principal component analysis was used to explore the global clustering of the samples. Pathway enrichment analyses were carried out with the MetaboAnalyst 5.0 tool [62] to explore human-relevant affected metabolic pathways against the KEGG database [63]. The top outputted pathways by fold-enrichment were selected, and unadjusted *p*-values were used for exploratory analyses.

## 4. Concluding Remarks

In this work, through the comparative analysis of the metabolomic profiles of plasma and different tissues from autophagy-proficient (WT) and autophagy-deficient (*atg4b^−/−^*) mice, we have been able to describe for the first time the metabolic footprint of a systemic autophagy inhibition (but not its total impairment) in laboratory mice (*atg4b^−/−^*). We have shown than the observed impact of autophagy deficiency in the metabolome is highly tissue-dependent, being much more accentuated in skeletal muscle than in heart, and less evident in liver tissue. In this regard, it might be argued that ATG4B deficiency could impact autophagy in a tissue-dependent manner. Due to possible variations in the expression levels of genes of other members of the ATG4 family, ATG4B deficiency could be differentially compensated by other ATG4 proteases according to their expression levels. However, the accumulation of p62 (a recognized marker of autophagy deficiency) upon ATG4B loss is comparable in heart, liver, and skeletal muscle tissues in mice [7], ruling out this possibility. Instead, the tissue-dependency of the observed metabolic alterations is more likely a consequence of the metabolic differences and requirements of each different tissue and also of the relative importance of autophagy in the context of tissue-specific metabolic reactions.

Our results show that the reduction in autophagic potential of leads to a reduction in the levels of different types of amino acids in skeletal muscle. This is likely a consequence of their reduced autophagic potential, given that this catabolic pathway is one of the main cellular amino acid suppliers [64]. This decrease in amino acid levels may impact skeletal muscle physiology, predisposing autophagy-deficient mice to develop muscle-related pathologies and probably rendering them more susceptible to suffer from age-related muscle atrophy. In this sense, the alterations observed in the lipidic profile and the reduction of GSH levels in autophagy-deficient muscles are similar to those observed in aged rodents, in which skeletal muscle atrophy is common. This is consistent with the proposed role for autophagy as an anti-aging process that helps to maintain tissue integrity through cellular self-renewal and provides energy and basic biomolecules for anabolic purposes.

In a similar way, most of the alterations observed in hearts from *atg4b^−/−^* mice are also associated with aging or aging-related pathologies. ATG4B deficiency leads to metabolic changes that increase the levels of several nitrogenous bases and nucleosides that are often observed either in cardiomyopathies or upon cardiac damage in laboratory rodents. Interestingly, the observed increase in the content of certain fatty acids and that of the methyltransferases inhibitor S-adenosylhomocysteine is associated with cardiac remodeling during aging, which is in line with some of the metabolic alterations found in skeletal muscle. Although the metabolic changes elicited by ATG4B deficiency in liver tissue are minor, we could also detect an increase in fatty acid content and in the metabolism of several fatty acids, which was also found increased in skeletal muscle and heart. This more general effect likely derives from the role of autophagic degradation in lipid droplet degradation [65].

In plasma, *atg4b^−/−^* mice showed an increase in the circulating levels of many amino acids, normally associated with metabolic disturbances and obesity. Thus, these alterations may help to explain why autophagy-deficient mice are usually more prone to the development of obesity and obesity-associated pathologies, such as type I and type II diabetes and metabolic syndrome [44]. Moreover, this increase in circulating amino acids not only involved all BCAAs, but also some of their keto acids (BCKAs). This resembles to some extent the metabolic alterations found in patients suffering from MSUD, a serious metabolic disorder that might be fatal when untreated with metabolic and dietary strategies. The fact that at least keto-isocaproic acid is able to inhibit autophagy in vivo [47] suggests that autophagy inhibition by elevated circulating BCCAs and BCKAs might contribute to the development of some of the detrimental symptoms of MSUD.

**Figure 7:**
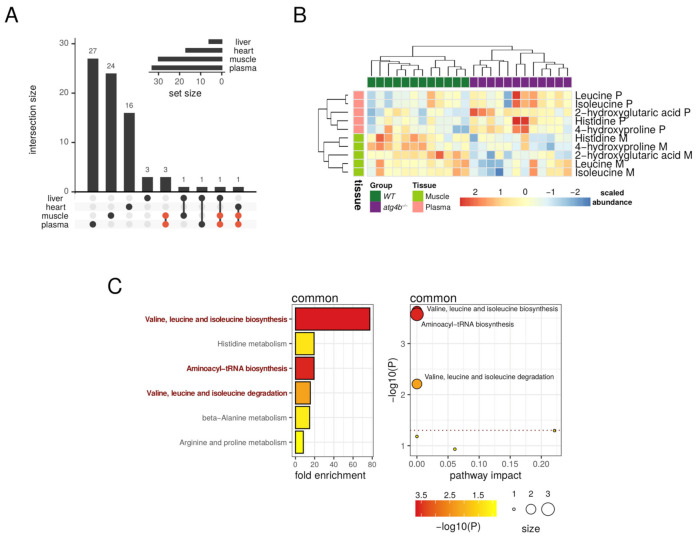
Oppositely targeted metabolites in plasma and muscle. (**A**) Upset plot showing the intersections of the sets of significantly altered metabolites found for each tissue. The five common metabolites between plasma and muscle are highlighted in red. (**B**) Heatmap describing the row-scaled log2 quantification levels of the five common metabolites detected as significantly altered in both plasma and muscle tissues. (**C**) On the left, the bar plot describes altered metabolic pathways with detected fold-enrichment, colored by significance (−log10 of *p*-value) for the five common metabolites detected as significantly altered in both plasma and muscle tissues. On the right, the scatterplot describes the pathway impact, size, and significance of the detected metabolic pathways. The horizontal dotted line in the scatterplot marks the 0.05 *p*-value threshold (unadjusted). Representative significant (unadjusted *p*-value < 0.05) pathways are highlighted in the bar plot and labeled in the scatterplot.

It is remarkable that the metabolic alterations caused by autophagy deficiency were mostly tissue-dependent, as we did not find any differential metabolite common to all the tissues analyzed (Figure 7A). However, five differentially altered metabolites (leucine, isoleucine, histidine, 4-hydroxyproline, and 2-hydroxyglutarate) were common to plasma and muscle (Figure 7A). Interestingly, all these metabolites were increased in *atg4b^−/−^* mice plasma samples as compared to WT mice, whereas they were reduced in mutant mice skeletal muscle samples. These results point out that autophagy deficiency leads to systemic alterations in metabolic pathways important for BCAAs metabolism and aminoacyl-tRNA biosynthesis (Figure 7B,C). However, autophagy deficiency affects these pathways in a divergent fashion in different tissues.

Taken together, the presented results show the impact of systemic autophagy inhibition in the metabolome of plasma and different tissues and might help to shed some light on the metabolic consequences of autophagy disruption in vivo. In some cases, we have found results that are at odds with those published using mice models with a total autophagy impairment (which is normally restricted to a tissue or cell lineage). In this regard, it should be pointed out that partial autophagy incompetency, such as that of *atg4b^−/−^* mice, is undoubtedly closer to a possible pathological situation involving autophagy inhibition (i.e., due to aging, poisoning with lysosomotropic agents, or infectious diseases) than a total autophagy impairment. Thus, our results might contribute to improving our understanding of how autophagic activity modulates metabolism in vivo.

## Data Availability

The data presented in this study are available in Supplementary Materials.

## Supplementary Materials

The following are available online at https://www.mdpi.com/article/10.3390/metabo11080481/s1. Table S1: Results of the differential metabolite analyses across tissues. The tables describe the list of metabolites, statistics (*p*-values, group means) and annotations (HMDB, KEGG IDs).
